# Proof of concept pilot study: prevalence of grass virus infection and the potential for effects on the allergenic potency of pollen

**DOI:** 10.1186/1476-069X-8-S1-S10

**Published:** 2009-12-21

**Authors:** Denise W Pallett, Emily Soh, Mary-Lou Edwards, Kathleen Bodey, Laurie CK Lau, J Ian Cooper, Peter H Howarth, Andrew F Walls, Hui Wang

**Affiliations:** 1 NERC/Centre for Ecology and Hydrology Oxford, Mansfield Road, Oxford, OX1 3SR, UK; 2 Infection Inflammation and Immunity Division, Mailpoint 837, Southampton General Hospital, Southampton SO16 6YD, UK

## Abstract

**Background:**

Wild plants harbour a variety of viruses and these have the potential to alter the composition of pollen. The potential consequences of virus infection of grasses on pollen-induced allergic disease are not known.

**Methods:**

We have collected pollen from *Dactylis glomerata *(cocksfoot; a grass species implicated as a trigger of allergic rhino-conjunctivitis) from Wytham Wood, Oxfordshire UK. Extracts were prepared from pollen from uninfected grass, and from grass naturally infected by the *Cocksfoot streak potyvirus *(CSV). Preparations of pollen from virus-infected and non-infected grasses were employed in skin testing 15 grass pollen-allergic subjects with hayfever. Allergen profiles of extracts were investigated by Western blotting for IgE with sera from allergic subjects.

**Results:**

The prevalence of CSV infection in cocksfoot grasses sampled from the study site varied significantly over an eight-year period, but infection rates of up to 70% were detected. Virus infection was associated with small alterations in the quantities of pollen proteins detected by polyacrylamide gel electrophoresis, and in the patterns of allergens identified by Western blotting with IgE from grass pollen allergic subjects. For individual subjects there were differences in potencies of standardised extracts of pollen from virus-free and virus-infected plants as assessed by skin testing, though a consistent pattern was not established for the group of 15 subjects.

**Conclusion:**

Infection rates for CSV in cocksfoot grass can be high, though variable. Virus-induced alterations in components of grass pollen have the potential to alter the allergenic potency.

## Background

Changes in the environment over the last half century have been associated with substantial increases in the prevalence of allergic disease, and particularly of allergic rhinitis and conjunctivitis. Allergic diseases now represent a major health care burden, with 35% of adolescents experiencing asthma-like symptoms, and approximately 25% of the population experiencing rhinitis at some time in their lives in the UK [1]. The loss of grassland due to urbanization and changing agricultural practice may be balanced by a reduction in allergy protective factors, such as early life exposure to infection and in particular, bacterial products. Environmental factors that can influence the potential of pollen to provoke allergic reactions deserve investigation.

Pollen from cocksfoot (*Dactylis glomerata*) is important as a source of allergens that provoke hayfever in the UK [2]. Cocksfoot is susceptible to many grass-infecting viruses. A natural cocksfoot population in Wytham Wood [3], Oxfordshire, UK, has been monitored for the *Cocksfoot steak virus *(CSV; genus, *Potyvirus*) prevalence since 2001. The viral infection triggers the grass anti-virus gene silencing, but the infection can persist for more than three years in infected grasses at glasshouse conditions [4].

Infections in plants have been reported to trigger the production of families of pathogen related proteins that mediate systemic acquired resistance for self-defence [5]. Viral infection of grasses can lead to expression of new antigens on the surface of pollen grains [6] and would be expected to lead to changes in the composition of allergens that are normally present. Infection by viruses has been shown to increase defence-related proteins and experimental studies on vegetables and trees have shown that upregulation of such proteins may not only increase the allergen content but also has the potential to increase allergenicity through cross reactivity [7,8]. A number of pathogen-related proteins in pollen extracts have been identified as allergens [9].

Allergic sensitivity to pollen and other allergens involves the production of immunoglobulin E (IgE) antibody in affected subjects. The binding of allergens to specific IgE antibody attached to membrane receptors on mast cells and basophils can trigger the explosive release of potent mediators of inflammation. Measurement of the binding of IgE to allergens, and experimental provocation of mast cell activation (e.g. by pricking allergen into the skin) provides a means for assessing allergenic potency.

The purpose of the present pilot study has been to generate prevalence data for CSV infection in cocksfoot, and to examine if virus infection may be associated with alterations in the allergenic potency of pollen from infected plants.

## Methods

Leaves were collected from individual cocksfoot plants in the Yellow Ants Reserve, Wytham Hill, Wytham Wood [3], Oxfordshire, in April-May 2001-2008. Leaf extracts were examined by Enzyme-Linked ImmunoSorbent Assay for CSV (ELISA, pre-2004) then by Reverse Transcription Polymerase Chain Reaction (RT-PCR, post-2004). In 2008, leaf samples were collected from 280 plants (79 from the Yollow Ants Reserve for virus infection rate, and the others were wild plants maintained at glasshouse after outdoor conditions during winter, for allergen test) before the pollen season. Total RNA was isolated from 0.2 g of leaf for each individual sample using the RNeasy Plant Mini Kit (Qiagen, Crawley, UK). CSV infection was determined from 1 μl of the RNA extract by using the One-step RT-PCR kit (Qiagen) with degenerated primers based on the CSV Nib cistron sequence [4] (forward, 5'-TCNCGNGARAARNGNAARTGG-3'; reverse, 5'-CNCCNGCRTTCATNGTYTG-3'; R = A/G, Y = C/T, N = A/G/C/T; GenBank accession No, EU119422, nt 6990-8577). A programme (50°C 30 min, 95°C 15 min, 30 cycles of 50°C 1 min, 94°C 1 min, 72°C 4 min, and finish with 72°C 10 min) was used to produce a CSV specific DNA band of 1587 bp in agarose gel electrophoresis. The infection rates were compared (number of positive vs number of negative) for different years using the Chi-Square test (Minitab 15).

Pollen was collected for each individual plant and stored at -80°C before use. Individual pollen extracts from infected (n = 10) and non-infected grasses (n = 8) were examined by light microscopy and incubated (1/10; w/v) with phosphate buffered saline (PBS, pH 7.4) at 20°C for 6 h. Extracts were centrifuged (200 ×g), filtered (0.22 μm) and protein concentrations determined by the Coomassie blue dye binding procedure with bovine serum albumin as the standard (Thermo, Cramlington, UK). Extracts from infected and non-infected plants (14 μl each) were subjected to sodium dodecyl sulphate (SDS) polyacrylamide gel electrophoresis (PAGE; 10%), and stained with Coomassie blue dye. Separate extracts from infected or non-infected grasses were pooled, and the protein concentration confirmed to be similar.

Subjects (n = 15) with a history of seasonal allergic rhino-conjunctivitis were recruited outside the pollen season from an advertisement placed in Southampton General Hospital. The study was approved by Southampton and South West Hampshire Local Research Ethics Committee. Skin prick tests were performed with serially diluted extracts of infected and non-infected pollen (neat, 1/10, 1/100, 1/1,000, 1/10,000) on the volar aspect of the forearm, and perpendicular diameters of the weal reaction determined. Serum was collected and incubated (diluted 1 in 10) with nitrocellulose blots of PAGE separated extracts. IgE-binding proteins were identified by sequential addition of biotinylated rabbit antibody specific for human IgE (Vector, Peterborough, UK), Extravidin peroxidase complexes (Sigma, Poole, UK) and Super Signal West Pico chemiluminescent substrate (Thermo).

## Results

**Figure 1:**
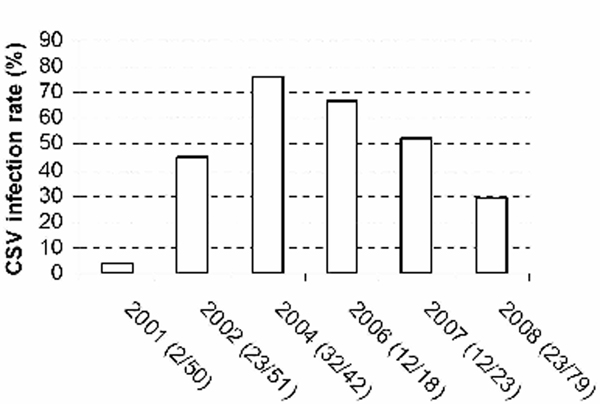
**CSV prevalence in cocksfoot at the Yellow Ants Reserve, Wytham Wood, Oxford**. Infection was deemed by readings greater than two times higher than negative controls in ELISA (2001-2004), and by positive RT-PCR amplification of the CSV Nib cistron (2004-2008). The infection rate is presented as a percentage, and actual numbers (positive/total) are shown for each year.

Infection rates in the natural cocksfoot population in the Yellow Ants Reserve, Wytham Wood were variable between 2001-2008 (Figure 1, Chi-Sq = 61.343, DF = 5, *P *< 0.001). The highest infection rate was more than 70% in 2004 when both ELISA and RT-PCR methods produced results conforming to each other. Since then, the infection rate had declined to just below 30% in 2008 (Figure 1, 2004-2008, Chi-Sq = 27.270, DF = 3, *P *< 0.001).

**Figure 2:**
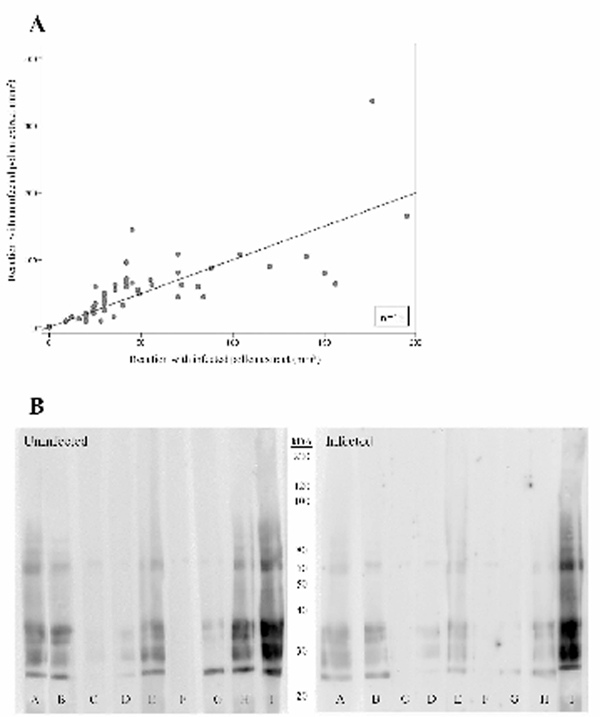
**(A) Relative sizes of skin prick test weals with extracts of pollen from uninfected and CSV-infected grasses**. Data is shown for five concentrations of each extract in 15 subjects. (B) IgE binding constituents identified by Western blotting of the uninfected (left) and virus-infected extracts (right) separated by PAGE. Results are shown for nine separate subjects, designated A to I.

Weal reactions to extracts from both infected and non-infected plants were elicited in all grass pollen-allergic subjects. There were differences in sizes of skin reactions between the extracts for individual subjects, though this was not apparent for the group as a whole (Figure 2A). Analysis of pollen extracts by SDS PAGE revealed similar patterns for virus-infected and non-infected plants, with quantitative differences more apparent than qualitative differences in components (not illustrated). Western blotting to identify IgE binding components in pooled extracts of pollen revealed considerable heterogeneity in responses of different subjects, with IgE from each subject binding to a different spectrum of proteins in the same pollen extracts. (Figure 2B). Comparison of IgE-binding patterns from each hayfever subject tested indicated differences between extracts of pollen from virus-infected and non-infected plants. For several subjects (e.g. A, B, E and H, Fig 2B) there was more intense staining of proteins of 25 to 38 kDa in pollen from uninfected grasses) than in that from the infected grasses. In contrast, for a protein with a molecular weight of approximately 65 kDa, there appeared to be more intense staining in the extract from infected grasses for some subjects (e.g., D, F, Figure 2B) but not others (e.g., A, C and G, Figure 2B).

## Discussion

A larger scale survey on CSV prevalence in the UK would be appropriate to reveal its impact on grassland ecology, crop production, and human hayfever. However, our studies have indicated that CSV infection can be present in a substantial proportion of the cocksfoot population growing in its natural habitat in Wytham Wood. It was striking the extent to which the prevalence varied from year to year in the natural reservation area sampled (Figure 1). Many factors are likely to contribute to the variation, and it is likely to be associated with the abundance of insect vectors (e.g., aphids for CSV). Long term trends are difficult to predict, but as plant immunity (gene silencing, and systemic acquired resistance pathways) is temperature dependent, the dynamics of plant-pathogen interactions and relationships may be expected to shift during the course of climate change.

A number of factors are likely to contribute to the allergenic potency of pollen. Of crucial importance are the ability to provoke the activation of mast cells in the tissues (here assessed by the measurement of skin test responses, Figure 2A), and the extent to which IgE responses can be elicited (examined by Western blotting for IgE with serum from allergic subjects, Figure 2B). In the present studies we found that there were differences in the size of skin weal responses elicited by the standardized extracts of pollens from infected and non-infected grasses. For the group of 15 grass pollen allergic subjects with hayfever, however, there was not a consistent trend for skin reactions to be greater for either extract (Figure 2A). Investigation of IgE binding of protein constituents of pollen extracts revealed differences in patterns between those from CSV infected and non-CSV infected plants. As with skin test responses there was a striking degree of variation between individuals (Figure 2B). Further studies are necessary, but it seems likely that some grass pollen allergic subjects may exhibit greater sensitivity to pollen from virus infected grasses, than to uninfected grasses, and vice versa. This has implications for the preparation of extracts for allergen immunotherapy and for diagnosis, and for an understanding of sensitization to pollens.

The precise nature of alterations in pollen from virus infected plants remains to be determined, and also the extent to which they may contribute to sensitization and the provocation of allergic reactions. The potency of an allergen as a stimulus for provoking allergic reactions may be influenced not just by the capacity to bind IgE and activate mast cells, but also by various factors that could be regarded as non-immunological. Thus various major allergens from pollen [10-12] as well as from other sources [reviewed in [13]] have been identified as proteases or other enzymes. Certain proteases from allergens may facilitate entry of allergen through epithelial linings [reviewed in [13]], or could stimulate generation of inflammatory mediators [10] and the activation of various cell types through protease activated receptors [14], or could participate in other inflammatory processes to augment allergic reactions. Moreover, eicosanoids released from pollen have been reported to have various pro-inflammatory actions [15-17], and lectins present could stimulate directly the release of mediators from mast cells and basophils [18].

## Conclusion

Viral and other pathogens naturally infect plants in the environment. Although these plant pathogens do not infect humans by themselves, their ability to alter the plant metabolism and to introduce new components into plants may have an impact on human health. Virus infection of grasses deserves consideration as a factor in pollen-induced allergic disease.

## List of abbreviations used

(CSV): *Cocksfoot steak virus*; (PAGE): Polyacrylamide gel electrophoresis; (ELISA): Enzyme-linked immunosorbent assay; (RT-PCR): Reverse transcription polymerase chain reaction; (SDS): Sodium dodecyl sulphate.

## Note

The peer review of this article can be found in Additional file 1.

## Competing interests

The authors declare that they have no competing interests.

## Authors' contributions

DWP, ES, EME and KB performed the experiments and analyzed data. JIC developed the hypothesis and collected grasses. AFW, PH and HW developed the hypothesis, and AFW and HW supervised studies, analyzed data, wrote the manuscript

## Supplementary Material

Additional file 1 Peer review Click here for file
