# Supplementary material for: Proof of concept pilot study: prevalence of grass virus infection and the potential for effects on the allergenic potency of pollen
Source: Environ Health. 2009 Dec 21;8(Suppl 1):S10. doi: 10.1186/1476-069X-8-S1-S10 (PMC2796488; doi:10.1186/1476-069X-8-S1-S10)
Supplement: Additional file 1 — Peer review [file 1476-069X-8-S1-S10-S1.doc]

| Article ref no. | A1471-2105X SZ-S10 |
| --- | --- |
| Title | **Grass virus infection: Prevalence and the potential for effects on the allergenic potency of pollens.** |
| Author(s) | Pallet et al. |

**Ref#1**

**General comments:**

A very interesting hypothesis, but the conclusions from the study would be more clearly drawn were the results more thoroughly presented and discussed.

Line numbering the manuscript would have helped the review.

***Response:***

Line numbers has been added in the manuscript.

**Major compulsory revisions:**

Need to see a fuller discussion of the results, especially the differences between the IgE binding of the pollen extracts, and the individual differences in reaction to infected and uninfected plant extracts by the test subjects.

***Response:***

The results section has been re-written and specific differences in the degree of IgE binding to pollen components are now highlighted.

**Minor essential revisions:**

Page 6, line 7: What was the size of the samples used for RNA extraction, e.g. how many mgs of leaf tissue was used? What was the volume of extract used as template in the RTPCR reaction? What was the actual name of the plant RNA extraction kit used?

***Response:***

The relative sentences have been changed to: Total RNA was isolated from 0.2 g of leaf for each individual sample using the RNeasy Plant Mini Kit (Qiagen, Crawley, UK). CSV infection was determined from 1 µl of the RNA extract by…

Comment:

P6, L10-11: Was the primer set developed for this study, or has it been used in previous work?

***Response:***

The primer set had been used previously for determination of the genome sequence of the local CSV strain (GenBank accession No, EU119422). We have added a reference citation as “…with degenerated primers based on the CSV Nib cistron sequence [4]…”

Comment:

P6, l14: change “Agarose” to “agarose”.

P6, l14-15: This sentence belongs at the start of the next paragraph.

***Response:***

Both changed.

Comment:

P6, L16: How were the pollen extracts prepared?

***Response:***

Further information on the preparation of extracts has been provided.

Comment:

P7, l 14: change “was” to “were”.

P7, l18: change “Weal reactions to both the infected and non-infected extracts” to “Weal reactions to extracts from both infected and non-infected plants”.

***Response:***

Both changed.

Comment:

P8, l5-8: “careful comparison….indicated..differences..”. Please describe these differences exactly. Looking at the figure it is difficult to see any differences, so please say where they are on the gel.

***Response:***

Specific differences in IgE binding to specific pollen components are highlighted for serum from selected subjects.

Comment:

P8, l30-31: Showing the results from the individual subjects and discussing them would help the conclusions of the paper.

***Response:***

Space constraints must limit the extent to which full analysis can be presented for all 15 of the allergic subjects tested. However, attention is drawn to some of the more prominent differences between individual subjects.

Comment:

Figure 2 A: Would it not have been clearer to have made one of the axes “extract concentration” and given separate symbols for extracts of infected and uninfected plants?

***Response:***

We have explored several means of presenting the skin test data graphically. On account of the large amount of data generated (15 subjects each tested with five different concentrations of two different extracts), we felt it better to present the results on a single graph which facilitated a direct comparison between skin responses to the two pollen extracts being tested (the potency of each being compared at the same concentration).

**Ref#2**

General comments.

This paper reports a small scale study on the interesting topic of the relationship between virus infection in grasses and the allergenicity of pollen. The question posed by the authors is fairly novel and is set in the context of some directly relevant similar work. The study design is of a small scale project from a very limited geographical area with relatively small sample sizes, both for the grasses and the testing of extracts of the pollen. As such this is an illustrative pilot study rather than comprehensive research. Many aspects of the paper need to be amended before it could be considered for publication.

***Response:***

We have changed the title to: “Proof of concept pilot study: prevalence of grass virus infection and the potential for effects on the allergenic potency of pollen”. Many more revisions have been made accordingly to the referee’s concerns.

- Major Compulsory Revisions (which the author must respond to before a decision on publication can be reached)

Comment:

The title needs to be amended to indicate the small region studied and the scale of the work

***Response:***

The title has been changed to: Proof of concept pilot study: prevalence of grass virus infection and the potential for effects on the allergenic potency of pollen.

Comment:

The stated purpose of the present study was to “ generate prevalence data for CSV infection of cocksfoot and to investigate the extent to which the composition of allergens or allergenic potency may be altered in pollen from infected plants.” This being the case the authors need to explain why plants from just one small area were used rather than samples from a wider geographical spread? Similarly, the amount of analysis of the allergens and the scale of the testing of reactions to extracts from the pollen used for skin prick testing are not able to provide much data to answer this question. The title suggests a much larger study than the one undertaken. The project design does not fulfill the stated purpose.

***Response:***

We have revised the last paragraph Page-5 to: The purpose of the present pilot study has been to generate prevalence data for CSV infection in cocksfoot, and to examine if virus infection may be associated with alterations in the allergenic potency of pollen from infected plants.

Comment:

More detail is needed to explain all aspects of the methods and to add rigor to the paper. For example, sample sizes are not justified either for the plants or the subjects (how were these sample numbers calculated?). Why was this virus chosen? Why were samples taken only from Wytham Wood and not other areas/ regions as well for comparison? What was the sampling scheme within the wood. Was it the same every year? Do other viruses also alter allergenicity of pollen ? Why was sampling of grass leaves done in April to May? How exactly was this done? How was random sampling achieved?

***Response:***

To address the referee’s concerns on sample numbers, we added a Chi-Sq test to statistically determine whether or not the infection rates were variable over the years. In the Methods, we added “The infection rates were compared (number of positive vs number of negative) for different years using the Chi-Square test (Minitab 15)” (Page 6). In the Results, we revised the first sentence to “Infection rates in the natural cocksfoot population in the Yellow Ants Reserve, Wytham Wood were significantly variable among 2001-2008 (Fig 1, Chi-Sq = 61.343, DF = 5, P < 0.001)”. In the figure legend of Fig.1, we added “The infection rate is presented as a percentage, and actual numbers (positive/total) are shown for each year”.

We are limited by 1500 words in the main text of this special issue. The virus was chosen for the pilot study because of the epidemic at the site in 2004, and that CSV is a member of the largest genus of plant viruses, the Potyvirus that infects both monocots and dicots. The Wytham Wood has been managed by the University of Oxford and the research site for many ecological studies. We added a citation of a recent publication ([3] Mihok B, Kenderes K, Kirby KJ, Paviour-Smith K, Elbourn CA: Forty-year changes in the canopy and the understorey in Wytham Woods. Forestry 2009, doi:10.1093/forestry/cpp021) in which site background information including site map is available.

Samples were taken in spring time because of a commonly accepted assumption that infections detected are likely being persistent/systemic rather than temporary/local infections when insect vectors become abundant. Sampling has been done in a random manner, and from distinguishable individual plants rather than grass clusters.

We appreciate the referee’s questioning on other viruses. As this pilot study shows the potyvirus (CSV) infection may change allergenic potency in grass (cocksfoot) pollen, large scale investigation is needed, including in other virus/plant associations.

Comment:

There is no demographic detail or allergy profile (other than that they were grass sensitive) for the 15 subjects used for the skin prick tests. These are required in order to assess the relevance of the results.

***Response:***

Mention is now made that all subjects suffered from allergic rhino-conjunctivitis, and the means and location at which they were recruited is now indicated. The terms of our local research ethics approval did not permit the collection of other data.

Comment:

Different sample sizes have been used for the grasses each year making cross comparisons (given in percentages) difficult.

***Response:***

A Chi-Square test has been added to show that the difference on infection rate of different years was statistically significant. In the Methods, we added “The infection rates were compared (number of positive vs number of negative) among years by using the Chi-Square test (Minitab 15)” (Page 6). In the Results, we revised the first sentence to “Infection rates in the natural cocksfoot population in the Yellow Ants Reserve, Wytham Wood were significantly variable among 2001-2008 (Fig 1, Chi-Sq = 61.343, DF = 5, P < 0.001)”. In the figure legend of Fig.1, we added “Infection rate is represented by percentage with actual numbers (positive/total) shown for each year”.

Comment:

The discussion lacks appraisal of the study and does not consider the results in full. Further discussion is needed on the possible reasons why the outcomes are inconclusive.

***Response:***

We have re-written parts of the Discussion in line with the comments made. We feel that the results are conclusive in providing information on viral (CSV) prevalence over a period of several years, and in indicating that there is a variable infection rate. We have also provided compelling data that the allergenic potency of pollen may be associated with CSV infection in grasses. Fig. 2 shows that the allergenicity of the infected pollen and non-infected pollen extracts were quite different for several of the subjects. There are of course a number of broader issues that arise from these findings and which are deserving of further study in the future.

Specific comments

Throughout the manuscript “pollens” needs to be changed to pollen. There is no plural.

***Response:***

Changed

Abstract

Results section

Comment:

Needs to state that a small geographical area was studied and to say where it was etc.

Second sentence; needs clarification “ proteins detected” ; from where in what?

***Response:***

This section has been revised to:

The prevalence of CSV infection in cocksfoot grasses sampled from the study site at Wytham Wood, Oxfordshire, UK, significantly varied over a eight-year period, but infection rates up to 70% were detected. Virus infection was associated with small alterations in the quantities of pollen surface proteins detected by polyacrylamide gel electrophoresis, and in the patterns of allergens identified by Western blotting with IgE from grass pollen allergic subjects. For individual subjects there were differences in potencies of standardised extracts of pollen from virus-free and virus-infected plants as assessed by skin testing, though a consistent pattern was not established for the group of 15 subjects.

Main paper

Background

Comment:

What was the approximate size of the area from which Dactylis glomerata was collected and the general climate and growth conditions? Why use the wild population for the allergen part of the work? It would have been better to have used growth in controlled conditions in order to minimize the variance in allergen production on pollen due to environmental factors so that viral infection was the main variable.

Why was sampling done from some wild plants in the reserve and some from other wild plants (maintained at glass house or outdoor conditions) ?

***Response:***

In the revised version, we cited a recent paper that provide background information of the Wytham Wood ([3] Mihok B, Kenderes K, Kirby KJ, Paviour-Smith K, Elbourn CA: Forty-year changes in the canopy and the understorey in Wytham Woods. Forestry 2009, doi:10.1093/forestry/cpp021). We did not use the pollen of field grasses for allergen tests. Instead, we collected pollen from glasshouse maintained wild cocksfoot with or without a CSV naturally infection, for the allergen tests. All pollen providing grasses were individually tested for CSV infections before pollen was harvested. To make this point clear, we revised the sentence in Page 6 to “In 2008, leaf samples were collected from 280 plants (79 from the Yollow Ants Reserve for virus infection rate, and the others were wild plants maintained at glasshouse after outdoor conditions during winter, for allergen test) before the pollen season”.

Comment:

Page 6 line 2 “leave” should be “ leaf”

***Response:***

Changed

Comment:

Why were different number of plants sampled in different years?

***Response:***

The original sample target was set to 50. After CSV infection became abundant, and to save staff time etc., sample target was reduced to 20. In 2008, we aimed to collect 80 samples from the field following funding being awarded for this project. The increased sampling number did not change the trend of a decreasing infection rate. All the sample size supported reliable Chi-Sq statistics.

Methods

Comment:

These lack essential detail

For example, “Individual pollen extracts from infected n=10 and non infected grasses n=8were incubated.” How much pollen was used? Was it all fully mature or at what stage of development? Why were different numbers of grasses sampled? Why were these small numbers sampled for this?

***Response:***

Totally 201 glasshouse maintained grasses were tested for CSV infections and set up trials of pollen harvesting. Flower shoots were enveloped in transparency plastic bags and trapped pollen was stored in -80˚C before use. However, only 10 infected and 8 non-infected plants produced sufficient amount of pollen for protein extraction. The quantities of pollen obtained from each of these 18 plants differed, but as is indicated, a constant ratio of 1/10 (w/v) was maintained in each extraction.

Results

Comment:

This section is short. It lacks detail and clarity

Fig 1 results for CSV infection rates are given as percentages. As they are very different sample sizes each year ranging from 18 to 79, they are not comparable.

***Response:***

We revised Fig. 1 and now it shows both the percentage and fraction (positive/total). The positive and negative numbers enables Chi-Sq test that shows the difference on infection rates are statistically highly significant. This information has also been given in revised Results as “(Fig 1, Chi-Sq = 61.343, DF = 5, P < 0.001)”, and (Fig. 1, 2004-2008, Chi-Sq = 27.270, DF = 3, P < 0.001).

Comment:

Fig 2 A. Why are the axes for the reactions with infected and non infected pollen shown on different scales?. This distorts the apparent visual relationship.

***Response:***

The scale of the axes was selected on the basis of the values for skin test area obtained, and it provides better separation of the data points. A line representing the point at which allergenic potency would be equal for the two extracts has been added so as to help indicate the visual relationship.

Comment:

Page 8 line 5 More detail is needed on the “ careful comparison of IgE binding patterns” . What is meant exactly by “small but quite definite differences “? What is the possible significance of these?

***Response:***

As mentioned in the response to Referee 1, this section of the Results has been re-written to improve clarity and to provide specific examples to illustrate the points made.

Discussion

Comment:

First sentence page 8 line 8 needs to be qualified to make it clear that the results relate only to one small area. What is the justification for extrapolating this to a general statement? The results are inconclusive and need to be set in the context both of appraisal of the study and of future work.

***Response:***

We revised the beginning of Discussion to: “Larger scale survey on CSV prevalence in the UK would be appropriate to reveal its impacts on grassland ecology, crop production, and human hayfever. However, our studies have indicated that CSV infection can be present in a substantial proportion of the cocksfoot population growing in its natural habitat in Wytham Wood”.

- Discretionary Revisions (which are recommendations for improvement but which the author can choose to ignore)

Comment:

It would be useful to also place the study in the wider context of other factors that can alter the allergenicity of pollen from one species both temporally e.g. from year to year, and through the pollen season and spatially/geographically. These factors include temperature and /or drought stress, soil conditions and air pollution. The relative importance of the influence of viral infections could then be placed in the context of wider scenarios rather than viewed in isolation.

***Response:***

We appreciate the referee’s view that larger and wider study in this area is needed.
